# Dynamics of cytokine and antibody responses in community versus hospital SARS-CoV-2 infections

**DOI:** 10.3389/fimmu.2024.1468871

**Published:** 2024-11-22

**Authors:** Tulika Singh, Andrew N. Macintyre, Thomas W. Burke, Jack Anderson, Elizabeth Petzold, Erica L. Stover, Matthew J. French, Thomas H. Oguin, Todd Demarco, Micah T. McClain, Emily R. Ko, Lawrence P. Park, Thomas Denny, Gregory D. Sempowski, Christopher W. Woods

**Affiliations:** ^1^ Duke Human Vaccine Institute, Duke University School of Medicine, Durham, NC, United States; ^2^ Division of Infectious Diseases and Vaccinology, School of Public Health, University of California, Berkeley, Berkeley, CA, United States; ^3^ Duke Global Health Institute, Durham, NC, United States; ^4^ Division of Infectious Diseases, Department of Medicine, Duke University Medical Center, Durham, NC, United States; ^5^ Center for Infectious Disease Diagnostics and Innovation, Duke University, Durham, NC, United States; ^6^ Division of General Internal Medicine, Department of Medicine, Duke School of Medicine, Durham, NC, United States; ^7^ RTI International, Research Triangle Park, NC, United States

**Keywords:** cytokines, antibodies, SARS-CoV-2, dynamics, community

## Abstract

**Introduction:**

Dysregulated host cytokine responses to SARS-CoV-2 infection are a primary cause of progression to severe disease, whereas early neutralizing antibody responses are considered protective. However, there are gaps in understanding the early temporal dynamics of these immune responses, and the profile of productive immune responses generated by non-hospitalized people with mild infections in the community.

**Methods:**

Here we conducted a prospective cohort study of people with suspected infections/exposures in the US state of North Carolina, before vaccine availability. We recruited participants not only in hospitals/clinics, but also in their homes. With serial sampling, we compared virologic and immunologic factors in 258 community cases versus 114 hospital cases of COVID-19 to define factors associated with severity.

**Results:**

We found that high early neutralizing antibodies were associated with lower nasal viral load, but not protection from hospitalization. Cytokine responses were evaluated in 125 cases, with subsets at first versus second week of illness to assess for time-dependent trajectories. The hospital group demonstrated a higher magnitude of serum IL-6, IL-1R antagonist, IP-10, and MIG; prolonged upregulation of IL-17; and lesser downregulation of GROα, IL-1R antagonist, and MCP1, in comparison to the community group suggesting that these factors may contribute to immunopathology. In the second week of illness, 2-fold increases in IL-6, IL-1R antagonist, and IP-10 were associated with 2.2, 1.8, and 10-fold higher odds of hospitalization respectively, whereas a 2-fold increase in IL-10 was associated with 63% reduction in odds of hospitalization (p<0.05). Moreover, antibody responses at 3-6 months post mild SARS-CoV-2 infections in the community revealed long-lasting antiviral IgM and IgA antibodies as well as a stable set point of neutralizing antibodies that were not waning.

**Discussion:**

Our data provide valuable temporal cytokine benchmarks to track the progression of immunopathology in COVID-19 patients and guide improvements in immunotherapies.

## 1 Introduction

The rapid global spread of severe acute respiratory syndrome coronavirus 2 (SARS-CoV-2) through airborne and asymptomatic transmission combined with inequitable access to lifesaving interventions overwhelmed healthcare systems, hampered economic development, and worsened global poverty and hunger (1–4). The disease caused by SARS-CoV-2 is called coronavirus disease of 2019 (COVID-19) and is characterized by a spectrum of mild to severe respiratory illness, with progressive pathologies involving systemic inflammation, acute respiratory distress syndrome, disseminated coagulation, organ failure, and death (5–8). The virologic damage in the first week of infection is followed by a hyperinflammatory immunologic phase, which is thought to underlie the progressive development of these pathologies (9). While inflammatory and protective immune responses can develop on the scale of hours to days, there is a gap in understanding the early temporal dynamics that contribute toward severe outcomes.

Symptoms of SARS-CoV-2 typically arise within 4–6 days (10). During this initial phase of illness, the virus replicates in the airway and damages lung tissue leading to the release of cytokines to recruit immune cells into the antiviral response (9, 11–15). However, the roles of cytokines in protection versus pathology are complex because they regulate multiple biological functions, have distinct combined effects, and are functionally redundant (16). While cytokine signals can promote virus-specific immunity like antibodies and cytotoxic T cells within 7–14 days, they can also cause hyperinflammation and hypoxia, which worsens disease severity (17–20). Serum taken during severe disease has abnormally high levels of inflammatory cytokines compared to that of healthy people (21, 22). Hyperinflammation promotes COVID-19-related pathologies in several ways as follows: (A) increased recruitment of immune cells, which can infiltrate lung tissue and reduce lung gas exchange function; (B) activation of immune cells, which can non-specifically damage vital organs (23); (C) exhausted immune cell phenotypes that cannot effectively deliver antiviral immune responses (24); (D) hypercoagulation that can create clots that impair essential organ functions and even elicit sudden acute pathologies like stroke and myocardial infarction; and (E) positive feedback for more pro-inflammatory cytokine production, such that even high levels of anti-inflammatory molecules cannot effectively downregulate inflammation (14, 16, 25, 26). Indeed, in individuals who die from COVID-19, the median time to death is 5–8 days after hospitalization, which is within the hyperinflammatory phase of disease (18–20, 27). Thus, dysregulated host immune responses are a primary physiologic cause of mortality due to COVID-19.

In contrast, high levels of SARS-CoV-2 neutralizing antibodies are associated with protection from hospitalization and may mitigate cytokine-related hyperinflammation (28, 29). Antibodies that bind the receptor-binding domain of spike protein (S) can block the virus from infecting cells (28, 30, 31). Yet, a shortcoming is that this antibody response may rise to functional levels too late in the disease to mitigate severe outcomes (32). Moreover, neutralizing antibodies elicited in mild seasonal coronaviruses are known to be short-lived (33). While the majority of SARS-CoV-2 infections have been in the community, there is a gap in understanding whether these mild infections elicit early and lasting protective antibody responses (34, 35).

In this study, we examined the temporal dynamics of cytokine and antibody responses in 406 SARS-CoV-2 infections from a prospective cohort in the state of North Carolina in the United States. We compared the immune profile of 258 mild community-based infections to 114 hospitalized cases and tested the hypotheses that (1) distinct inflammatory responses are associated with hospitalization in the first versus second week of illness, (2) low early neutralizing antibody responses are associated with hospitalization, and (3) mild infections are associated with waning of neutralizing antibodies. Our data indicate that there are different networks of cytokines in the first versus the second week of illness, which are associated with hospitalization and four distinct phases of antibody waning after mild community-based infections.

## 2 Materials and methods

### 2.1 Ethics statement

Written consent was obtained from the participants or, in some cases their legally authorized representatives. Participation in these studies was voluntary. All research protocols were approved by the Duke University Health System Institutional Review Board (IRB) and performed in accordance with the Declaration of Helsinki. The IRB-approved protocol number for the Molecular and Epidemiological Study of Suspected Infection (MESSI) is Pro00100241. In addition, we utilized previously biobanked samples, which have the following study identifiers and IRB-approved protocol numbers: Validation of Clinical Molecular Predictors in Pre-symptomatic Infectious Diseases (Pro00001176) and the associated Biorepository protocol (Pro00001698).

### 2.2 Prospective cohort and sampling approach

We enrolled members of the community and hospital patients into the Molecular and Epidemiological Study of Suspected Infection (MESSI), an open prospective cohort study of infections in North Carolina in people aged 2 or older, as previously described (36, 37). The purpose of MESSI is to collect samples and data for research from individuals who have been exposed to someone with an infection, suspected to have an infection, or have been diagnosed with an infection, including COVID-19, to study biomarkers of infectious diseases and better diagnose infection. In this study, we included participants enrolled during the introductory pandemic of SARS-CoV-2 in North Carolina between March 2020 and November 2021. We used samples and clinical data collected during the pre-vaccination era in this observational study of natural infections. To our knowledge, the timepoints included in this study represent one infection event per person. We did not ascertain any SARS-CoV-2 infections that may have occurred prior to enrollment. Given that the study period was during the introductory pandemic of SARS-CoV-2 in North Carolina, with alpha and delta variants, it is likely that we sampled the first infection for most of our study participants, though this cannot be confirmed. Community-based participants were identified through recruitment at homes, a homeless shelter, skilled nursing facilities, hospice care facilities, and long-term care centers, whereas hospitalized participants were identified and recruited through the Duke University Health System (DUHS) or the Durham Veterans Affairs Health System (DVAHS). Upon written consent, participants provided symptom and demographic information, a nasal swab, and blood sample at enrollment. Subsequently, we followed up with participants for symptom information and sampling every few days in the first week, then weekly through the first month, and then at 2, 6, and 12 months post enrollment, where possible. This approach provided longitudinal blood samples and clinical information in the participants who consented to our follow up and were able to be reached. Time of symptom onset was ascertained by participant recall. In addition to this main study group, we used previously biobanked samples from other studies collected before 2020 (pre-SARS-CoV-2 pandemic) to generate comparator groups of cytokine responses in healthy individuals with no apparent infection (n = 18).

### 2.3 Classification of COVID-19 cases in the cohort

The presence of SARS-CoV-2 natural infection was defined by the following: (A) PCR positive in nasopharyngeal swab or plasma within 14 days of symptom onset or first visit and (B) positive result on blood test for viral antigen-binding IgM or IgG commercial tests (EUROIMMUN or Abbott IgG/IgM) within 21 days or symptom onset. Data on symptoms, severity of illness, and medications were collected from case report forms and review of the medical record. SARS-CoV-2 vaccinees were censored from this study at the date of first vaccine dose, and their data were excluded from that point onward. This allowed for controlled study of natural SARS-CoV-2 immunity. If a COVID-19 case was treated in the hospital at any point during a participant’s illness, we assigned that case to the Hospitalized analytic group, whereas those COVID-19 cases not treated in the hospital at any point during their illness were assigned to the Community analytic group.

### 2.4 SARS-CoV-2 quantification in nasal swab by qRT-PCR

Viral load in nasal swab was assessed as previously described (38, 39). Briefly, nasal swab viral transport medium (VTM) was aliquoted and cryopreserved from study subjects to determine SARS-CoV-2 N1 gene copy number by RT-PCR to stratify subjects such as COVID PCR positive or negative. Viral RNA was extracted from 140 µl of VTM according to manufacturer’s instructions (QiaAmp Viral RNA minikit). SARS-CoV-2 nucleocapsid (N1) and human RNase P (RPP30) RNA copies were determined using 5 µl of isolated RNA in the CDC-designed kit (CDC-006-00019, Revision: 03, Integrated DNA Technologies 2019-nCoV kit). Standard quantitative RT-PCR (TaqPath 1-step RT qPCR Master Mix, Thermofisher) was run with test RNA and gene-specific standard curves (2 x 10^5^–20 copy/ml). Regression analysis was used to determine gene copy number and corrected to report copies/ml of VTM. Samples with a Ct value greater than 35 PCR cycles were called SARS-CoV-2 PCR negative and samples less than or equal to 35 were called SARS-CoV-2 PCR positive.

### 2.5 SARS-CoV-2 “High Sensitivity” qPCR for plasma viremia

QIAGEN QIAsymphony DSP Virus/Pathogen Midi Kit (96-well QIAgility) was used for viral RNA extraction from 0.4 or 0.8 ml of plasma. The envelope gene of SARS-CoV-2 was reverse transcribed and amplified by PCR on an Applied Biosystems QuantStudio 3 Real-Tim PCR System. A standard curve was run with each batch to extrapolate RNA copies/ml. The lower limit of quantification (LLOQ) for this assay was 62 RNA copies per mL (1.79 log_10_) with 0.8 ml of plasma input or 128 RNA copies per ml with 0.4 ml of plasma diluted 1:1. Values below the LLOQ may be outside of the 95% confidence interval for reproducibility. Any positive SARS-CoV-2 viral load detected was considered “positive” and not detected was considered “negative.”

### 2.6 Serologic diagnostic testing by commercially available kits

Over the course of the early pandemic and this study, multiple kits were used to detect spike-binding IgG and IgM antibodies in blood samples. Human EDTA-plasma or serum were used for all assays.

#### 2.6.1 EUROIMMUN

An IgG antibody response toward the spike S1 domain (including receptor-binding domain, RBD) was tested with the anti-SARS-CoV-2 IgG ELISA assay (EUROIMMUN Medizinische Labordiagnostika AG) according to manufacturer’s instructions. Test results were evaluated by calculating the ratio of the OD (optical density) of the test sample over the OD of the calibrator sample. A ratio of <0.8 was interpreted as negative and a ratio of 1.1 or greater as positive (ratio of 0.8 to <1.1 as indeterminate and not utilized in phenotyping).

#### 2.6.2 Abbott qualitative

IgG antibody response against the SARS-CoV-2 nucleoprotein was measured using the SARS-CoV-2 IgG (Abbott) chemiluminescent microparticle immunoassay (CMIA) on the Abbott Alinity platform for qualitative detection of IgG. Qualitative results are reported as index values (S/C), where the relative fluorescence of sample was divided by relative fluorescence of calibrator. As per kit instructions, an S/C of 1.4 AU was considered positive, and <1.4 was considered negative. We also applied the AdviseDx SARS-CoV-2 IgG II (Abbott) CMIA on the Abbott Alinity platform) to detect IgG antibody responses against Spike RBD. For this, 50 arbitrary units (AU)/ml was considered positive, and <50 AU/ml was considered negative. Similarly, for qualitative detection of IgM antibodies against the SARS-CoV-2 RBD, the AdviseDx SARS-CoV-2 IgM (Abbott) CMIA was used on the Abbott Alinity platform. As per kit instructions, an S/C of 1.0 AU was considered positive, and <1.0 was considered negative.

### 2.7 Spike- and nucleoprotein-binding antibody

The magnitude of anti-spike and anti-nucleoprotein IgG, IgA, and IgM antibodies was assessed via ELISA. A total of 384-well high-binding plates (Corning) were coated for 2.5 h at room temperature (RT) with 2 μg/ml of recombinant SARS-CoV2 spike S2 Extracellular domain (Sino Biological 40590-v08b) or nucleocapsid protein (Sino Biological 40588-v08b) in CBC buffer (15 mM Na_2_CO_3_, 35 mM NaHCO_3_, pH 9.5; all Sigma). Antigen was removed by aspiration, and then plates were blocked overnight at 4°C in CBC, 5% goat serum, 0.01% Kathon, 0.05% Tween-20 (all Sigma), and washed in PBS (ScyTek) 0.1% Tween-20 (Sigma). Heat inactivated sera (1 h, 60°C) were diluted 1:30 in PBS, 5% goat serum, 1% milk, 0.05% Tween-20, 0.01% Kathon, threefold serially diluted to 1:143,489,070 in the same buffer and then applied to the antigen-coated, blocked plates. Plates were incubated overnight at 4°C, washed, and then horseradish peroxidase (HRP)-conjugated detection antibodies (goat anti-IgG, goat anti-IgM both Jackson Labs; goat anti-IgA from Southern Biotech) were applied at optimized dilutions in PBS, 10% goat serum, 0.05% Tween-20. Plates were incubated for 105 min at RT, washed, and then TMB-Ultra HRP substrate (Pierce) added. Plates were incubated for 14 min at room temperature and then the HRP reaction stopped by the addition of 2 N H_2_SO_4_ (Sigma). Plate absorbance at 450 nm was immediately read using a SpectraMax Plus plate reader (Molecular Devices). Area under the titration curve was determined using the trapezoid method using Microsoft Excel. All steps, except transfer to cold storage, were performed by a BioMek i7 liquid handler.

### 2.8 Quantifying cytokine levels in serum

Cytokines were assayed in duplicate using non-heat-inactivated undiluted serum with a commercial 47-plex bead array (Millipore HCYTA-60K-47C) performed according to the manufacturer’s instructions and read using a Flex-MAP 3D bead reader (Luminex). Data were analyzed using Bio-Plex manager v6.2. The 47 measured cytokines and chemokines included the following: interferon alpha (INF-α), interferon gamma (INF-γ), interleukin 1 alpha (IL-1α), interleukin 1 beta (IL-1β), interleukin 1 receptor antagonist (IL-1Ra), interleukin 2 (IL-2), interleukin 3 (IL-3), interleukin 4 (IL-4), interleukin 5 (IL-5), interleukin 6 (IL-6), interleukin 7 (IL-7), interleukin 8 (IL-8), interleukin 9 (IL-9), interleukin 10 (IL-10), interleukin 12 heterodimer p40 and p35 (IL-12p70), interleukin 12 homodimer p40 (IL-12p40), interleukin 13 (IL-13), interleukin 15 (IL-15), interleukin 17 alpha (IL-17α), interleukin 22 (IL-22), interleukin 27 (IL-27), interleukin 18 (IL-18), interleukin 17E (IL-17E), interleukin 17F (IL-17F), macrophage colony-stimulating factor (M-CSF), monokine induced by gamma (MIG), tumor necrosis factor alpha (TNF-α), epidermal growth factor (EGF), fibroblast growth factor (FGF), granulocyte colony-stimulating factor (GCSF), granulocyte–macrophage colony-stimulating factor (GM-CSF), monocyte chemotactic protein 1 (MCP-1), interferon gamma-induced protein 10 (IP-10), macrophage inflammatory protein 1 alpha (MIP-1α), macrophage inflammatory protein 1 beta (MIP-1β), tumor necrosis factor beta (TNF-β), vascular endothelial growth factor (VEGF), FMS-like tyrosine kinase 3 ligand (Flt-3L), fractalkine, growth-related protein (GROα), monocyte chemotactic protein-3 (MCP-3), macrophage derived chemokine (MDC), platelet derived growth factor AA dimer (PDGFa), platelet derived growth factor AB and BBdimer (PDGFab), soluble CD40 ligand (sCD40L), RANTES (CCL5), eotaxin, and transforming growth factor alpha (TGF-α).

### 2.9 Fluorescent SARS-CoV-2 microneutralization test

Vero E6 cells (ATCC CRL-1586) were seeded at 4 x 10^4^ cells/well in black 96-well plates in growth medium made of MEM + Earl’s salts + L-glutamine (Gibco 11095), 100 U/ml of penicillin and 100 µg/ml of streptomycin (Gibco 15140), 1 mM sodium pyruvate (Gibco 11360), 1× non-essential amino acids (Gibco 11140), and 10% fetal bovine serum (Gemini 100-106). Cells were allowed to adhere overnight. Serum samples were heat-inactivated for 1 h and diluted twofold in virus diluent, which is the same as the growth medium except with 2% fetal bovine serum instead of 10%. Each serum sample was incubated with 100 TCID50 fluorescent SARS-CoV-2 (40), strain USA/WA1/2020, for 1 h at 37°C. Following incubation, the serum/virus solution was added to Vero E6 cells and incubated at 37°C for 24 h. Then, cells were fixed using 10% neutral-buffered formalin and washed with PBS. Fluorescent signal was measured using a BioTek Synergy H1 microplate reader. Percent inhibition was calculated and used to determine IC_50_ values with non-linear regression analysis tools from GraphPad Prism. In each assay, samples were measured in duplicate alongside established positive and negative controls.

### 2.10 Logistic regression model development for cytokine responses

To identify a group of key cytokines with independent contributions toward hospitalization, each cytokine was evaluated in a stepwise manner to produce a minimal unbiased cytokine set related to outcome for each <6 DPOS and 8–12 DPOS periods. For all analyses, each cytokine (C_1_) was centered to the mean of log_2_(pg/ml) for the healthy group, and the reference was baseline odds for a 45-year-old male with mean level of cytokines as per the healthy group. First, each cytokine (C_1_) was regressed onto the outcome of hospitalization in a univariable regression model (Equation [Eq1] in **Supplementary Table S3**). Second, each cytokine (C_1_) with potential for independent contribution toward hospitalized from Eq 1 moved forward into Eq 2 and assessed in multivariable logistic regression model for significant contribution to hospitalization with adjustment for known confounders of age and sex (Eq 2; **Supplementary Table S3**). Third, Eq 2 was expanded to include the potential of age and sex as effect modifiers for each cytokine (Eq 3; **Supplementary Table S3**). Based on these relationships, independently contributing cytokines and their effect modifiers (p < 0.05 in any of the models of Eq 2 or 3) were tested together in a multivariable logistic regression to identify a minimal combination at each <6 and 8–12 DPOS, which optimally explains COVID-19 hospitalization. A top–down strategy was applied to build three models for each period, and the optimal model was selected on the basis of goodness of fit assessed with the lowest Akaike information criterion. All models included age and sex as known confounders. The final multivariable logistic regression model of cytokines for <6 DPOS was Equation 4 (**Supplementary Table S3**). The final multivariable logistic regression model of cytokines for 8–12 DPOS was Equation 5 (**Supplementary Table S3**).

### 2.11 Statistical models for the magnitude and kinetics of antibody responses

A generalized estimating equation (GEE) model was generated for SARS-CoV-2 neutralizing antibodies, and each binding antibody population was measured (n = 6): anti-spike IgG, IgM, and IgA; anti-nucleoprotein IgG, IgM, and IgA (**Supplementary Figure S3**). The outcome for each model was the level of antibody, where the magnitude of antibody was measured in terms of log_2_ of area under the curve, and neutralizing antibodies in terms of log_10_ of 50% maximal virus neutralizing titer (IC_50_). All models were adjusted for intercorrelation due to inclusion of multiple longitudinal measures from the same individual over time. Time bins for comparison were 0–20 (bin #1) and >20–40 (bin #2) DPOS, where interval 2 denotes the intercept for time bin 2. The reference group was <40 years old males among the community COVID-19 cases (Eq 6; **Supplementary Table S3**). To assess rates of change in antibody level over time among community COVID-19 cases, a generalized linear mixed effects model was applied by time bins: 0–20 (bin #1), >20–40 (bin #2), >40–100 (bin #3), and >100 (bin #4). Int1, 2, 3, and 4 define the intercepts for each of the aforementioned time bins. The reference group was 45-year-old males in the community (**Supplementary Figure S4**; Eq 7 in **Supplementary Table S3**). Data were analyzed in Stata and visualized with either Stata, GraphPad Prism, or Microsoft Excel.

## 3 Results

### 3.1 Prospective cohort of USA-based community versus hospital SARS-CoV-2 infections

In our observational cohort, we enrolled 639 participants from the community and hospital settings in the state of North Carolina, USA, and collected data on 2,736 person-visits from March 2020 through November 2021. Our main objective was to understand immune responses to natural infection in the community. Thus, 84% of the participants’ first visit occurred in the community: 67% at home and 17% in communal living conditions including hospice care, long-term acute care, skilled nursing facilities, or homeless shelters (**Supplementary Table S1**). During our observational study, the alpha, beta, and delta lineages of SARS-CoV-2 circulated in North Carolina, and we included timepoints before vaccine availability (41, 42). We found that 406 individuals were SARS-CoV-2 test positive at the time of enrollment (COVID-19 group), 67 had proven non-COVID-19 related illnesses, and 166 had an indeterminate diagnosis at the time of analysis (defined as clinical symptoms of possible infection without positive confirmatory testing, **Supplementary Table S1**; **Supplementary Figure S1**). The COVID-19 group includes 72 individuals whose diagnosis was confirmed through self-reported molecular or antibody tests. During our study period, more COVID-19 cases (29%) were treated in the hospital than in other etiologies (4%–10%; **Supplementary Table S1**).

From the COVID-19-positive participants (n = 406), we formed comparator groups of severe versus mild disease using hospitalization as a proxy for severity. We compared individuals treated in the hospital (n = 114, hospital group) versus not treated in the hospital (n = 258, community group) during their illness (**Table 1**; **Supplementary Figure S1**). Of note, 34 participants were excluded from analysis due to missing virologic or immunologic data. Mean age was significantly higher (54–60 years) in the hospitalized group compared to the community group (40-44 years; Wilcoxon signed-rank test, p < 0.05; **Table 1**). Sex was not significantly different by hospitalization (Fishers exact test; **Table 1**). The races of participants were significantly different across groups with fewer White participants and more Black participants in the hospitalized group than in the community group (Fishers exact test, p < 0.05; **Table 1**). Other races of participants included in this study were Asian (n = 20), Native Hawaiian-Pacific Islander (n = 1), “More than one” race (n = 22), American Indian-Alaska Native (n = 6), and unknown/unspecified (n = 6). Hispanic ethnicity was comparable across groups (14% versus 15%; Fishers exact test; **Table 1**). The median day of first visit was 11 and 8 days post symptom onset (DPOS) in the community and hospital groups, respectively (**Table 1**). At the first visit, 63% of all COVID-19 participants demonstrated detectable viral RNA (vRNA) in nasopharyngeal (NP) swab (**Table 1**). The NP swab viral load at first visit and peak within 20 DPOS (4.5–4.7 log_10_ vRNA copies/ml) were comparable across groups, as well as the timing of peak viral load on the ninth DPOS (Wilcoxon signed-rank test; **Table 1**). However, in line with our prior findings, a significantly greater portion of the hospital group demonstrated viremia in blood than the community group at first visit (27% versus 2%; Fishers exact test, p < 0.05; **Table 1**) (43). In the hospital group, 25% of the participants were admitted to the intensive care unit (ICU), 14% were intubated, and 9% died. Among our study participants, older age, viremia, and Black race were associated with severe disease as measured by hospitalization.

**Table 1:** Demographics and virologic characteristics of confirmed COVID-19 cases in the community versus hospitalized groups.

	Community (Non-hospitalized)	Hospitalized	*p*
**Participants (n)**	258	114	
**Observations (n)**	985	248	
**Mean Age at enrollment** **(95% confidence interval)**	42 (40-44)	57 (54-60)	<0.05
**Sex (% Female)**	48%	50%	ns
Race and Ethnicity			
White (n)	60% (154)	40% (45)	<0.05
Black (n)	24% (62)	49% (56)	<0.05
Other (n)	16% (42)	11% (13)	ns
**Hispanic ethnicity (n)**	14% (36)	15% (17)	ns
Outcomes and Follow up			
**Deaths % (n)**	0	9.65% (11/114)	<0.05
**Intubation % (n)**	NA	14% (16/114)	NA
**ICU Admission % (n)**	NA	25% (30/114)	NA
**Median days in hospital** **(min - max) n**	NA	6 (1-126) 104	NA
**Mean days of follow up per person (95% confidence interval) n**	47 (39-55) 208	9 (5-13) 94	<0.05
Participant location at first visit			
Acute care / Hospital setting (n)	3.1% (8)	52.2% (58)	<0.05
Homeless (n)	17% (44)	0	NA
Home (n)	79% (204)	26.1% (29)	<0.05
Hospice Care (n)	0	0.9% (1)	NA
Long term acute care (n)	0.4% (1)	0.9% (1)	ns
Skilled nursing facility (n)	0	17.1% (19)	NA
Other (n)	0.4% (1)	2.7% (3)	<0.05
**Median day since symptom onset at first visit (95% CI) (n)**	11 (16-22) (211)	8 (8-12) (95)	<0.05
**Spike IgM positivity at enrollment (n)**	52% (122/235)	64% (66/103)	ns
Viral Load			
**Nasopharyngeal swab N-protein PCR positivity at first vist (n)**	63% (152/241)	65% (37/57)	ns
**Detection of vRNA in plasma at first visit (n)**	2.4% (3/124)	27% (16/59)	<0.05
**Mean Log10 peak vRNA copies/mL in NP swab within 20 days of symptoms (95% CI) (n)**	4.7 (4.3-5.1) (99)	4.5 (3.8-5.2) (32)	ns
**Day of peak vRNA in NP swab within 20 days of symptoms (95% CI) (n)**	9.1 (8-10) (99)	9.5 (7-11) (32)	ns

ns means not significant.NA means not applicable.

### 3.2 Altered cytokines in COVID-19 cases relative to healthy individuals

**Figure 1:**
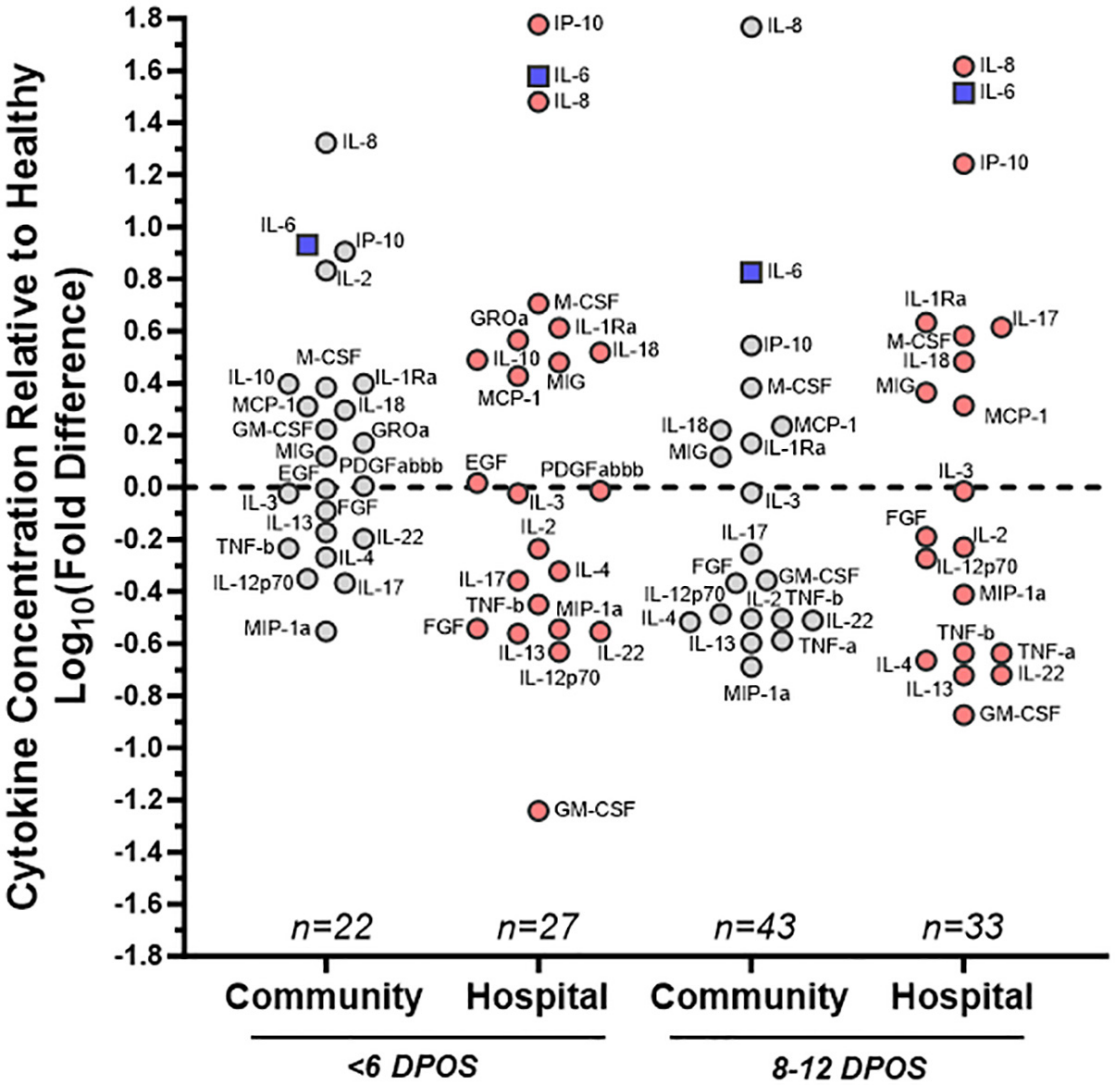
Distinct patterns of cytokine dysregulation in community versus hospital groups of SARS-CoV-2 infections at <6 and 8–12 DPOS relative to healthy individuals. Cytokine concentration (pg/ml) was measured via multiplex bead assay using sera collected <6 or 8–12 DPSO from cohort participants as well as healthy controls pre-pandemic. Mean levels of each cytokine, from subsets of community (gray) and hospital (red) COVID-19 cases, were assessed as a fold change relative to healthy control group. On log_10_ scale, 0 indicates no difference, positive values are increased concentration, and negative values are decreased concentration relative to the mean of healthy control individuals. Cytokines farthest from 0 were furthest from healthy. For reference, IL-6, a known pathological cytokine in COVID-19 is boxed in blue. Samples were assayed in duplicate and group size indicated on plot (n). Labels for each cytokine adjacent to each circle.

Using a 47-plex cytokine assay, we characterized early serum cytokines upon SARS-CoV-2 infection in different individuals from the first (<6 DPOS; n = 49) versus second week (8–12 DPOS; n = 76) of illness (**Supplementary Figure S1**). These time periods distinguish the virologic versus immunologic phases of pathology (13). As a reference group, we selected serum samples from 18 healthy (i.e., no acute illness or infection) North Carolina individuals collected before 2020 (i.e., pre-pandemic). Of the 47 cytokines measured, 24 were at significantly different serum concentrations (log_2_ pg/ml) in early SARS-CoV-2 infections compared to those of the healthy (Wilcoxon signed-rank test; p < 0.05). Of these, 19 were different at both time periods (FGF, GM-CSF, IL-1Ra, IL-2, IL-3, IL-4, IL-6, IL-8, IL-12p70, IL-13, IL-17, IL-18, IL-22, IP-10, MCP-1, M-CSF, MIG, MIP-1α, TNF-α), four were different only at <6 DPOS (EGF, PDGFABBB, IL-10, and GROα), and TNF-α was different only at 8–12 DPOS (**Figure 1** and **Table 2**). Thus, we used an unbiased approach to identify subsets of cytokines that were differentially expressed in COVID-19 in the first versus second week of illness.

**Table 2:** Mean serum concentrations of the 28 cytokines that were significantly different from healthy individuals in the community and hospitalized SARS-CoV-2 infection groups, and percentage changes within groups over time.

Cytokine name	<Day 6		Day 8-12			Healthy Mean (pg/mL)		Change in means within a group from <6 to 8-12 DPOS			
	COVID-19 Community Mean (pg/mL)	COVID-19 Hospital Mean (pg/mL)	COVID-19 Community Mean (pg/mL)		COVID-19 Hospital Mean (pg/mL)			COVID-19 Community (%)		COVID-19 Hospital (%)	
**EGF**	7.2	7.6	7.7		7.5	7.3		6.9		-1.3	
**FGF2**	330.8	117.4	175.0		263.5	408.0		-47.1		124.5	
**GM-CSF**	64.4	2.2	17.0		5.2	38.5		-73.6		133.3	
**GROa**	44.5	110.4	36.8		79.3	30.0		-17.4	*	-28.1	
**IL1Ra**	49.0	80.1	29.0		84.2	19.6		-40.8	*	5.1	
**IL-2**	15.7	1.3	0.7		1.4	2.3		-95.4		1.4	
**IL-3**	0.6	0.6	0.6		0.6	0.7		0.5		1.9	
**IL-4**	3.9	3.5	2.2		1.6	7.3		-43.6		-54.7	
**IL-6**	12.1	53.8	9.5		46.5	1.4		-21.5		-13.7	
**IL-8**	116.5	167.3	324.3		228.7	5.5		178.4		36.6	
**IL10**	29.4	36.4	12.5		23.6	11.8		-57.4		-35.3	
**IL12p70**	18.8	9.9	13.8		22.6	42.3		-26.6		128.7	
**IL-13**	113.2	46.2	42.6		32.0	168.3		-62.4		-30.7	
**IL-17**	3.3	3.4	4.3		31.8	7.7		29.7		840.2	
**IL-18**	58.8	98.1	49.3		90.4	29.7		-16.2		-7.9	
**IL-22**	107.5	47.1	52.2		32.3	168.9		-51.5		-31.5	
**IP10**	1068.5	7954.9	465.7		2318.1	132.7		-56.4	*	-70.9	*
**MCP1**	697.9	913.4	586.2		704.2	341.5		-16.0	*	-22.9	
**MCSF**	155.9	327.5	155.5		245.9	64.4		-0.2		-24.9	
**MIG**	2648.2	6075.1	2633.9		4657.2	2011.6		-0.5		-23.3	
**MIP1a**	28.4	28.9	20.8		39.3	101.2		-26.9		35.9	
**PDGFabbb**	26824.4	25770.3	35112.5		47416.8	26515.6		30.9		84.0	
**TNFa**	206.1	71.6	59.9		53.5	232.0		-70.9		-25.2	
**TNFb**	86.0	52.4	46.1		34.1	147.5		-46.4		-34.9	
***n individuals***	22	27	43		33	18					

	***Drop in Cytokine levels***				***Rise in cytokine levels***						
	>100%	>50%		>20%	0 +/- 20%		>20%	>50%		>100%	

* means significantly different.

### 3.3 Distinct trajectories of cytokine responses in relation to hospitalization

**Figure 2:**
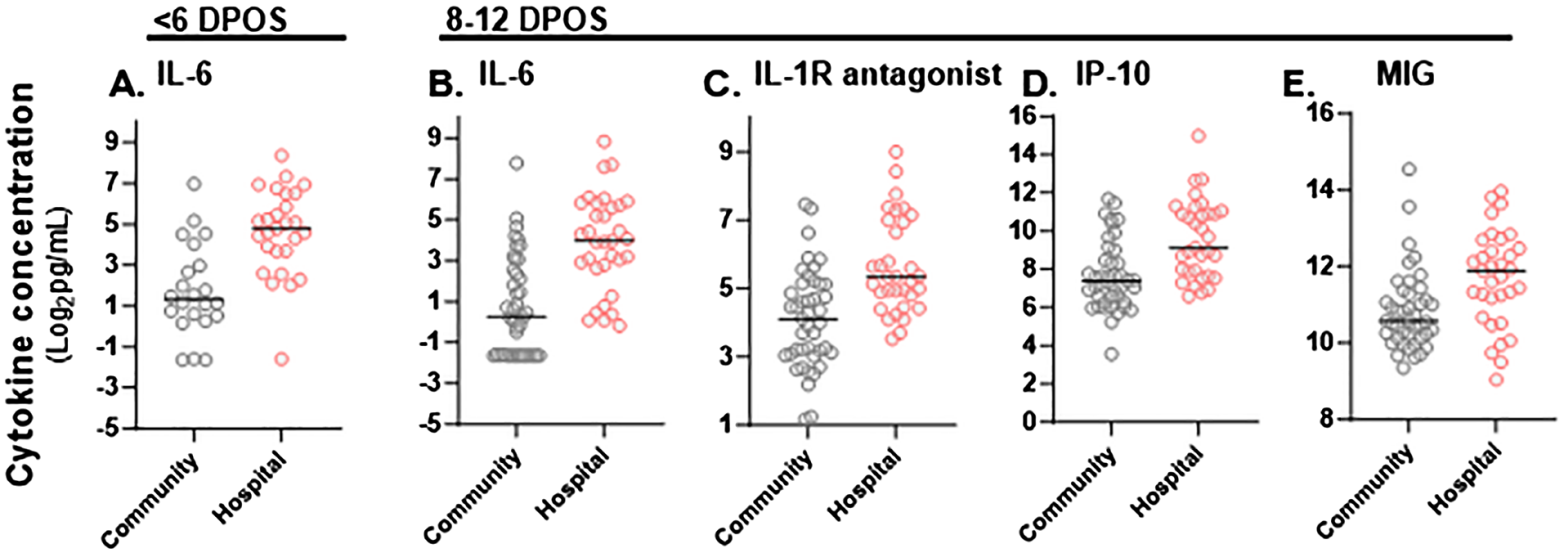
Significant differences in magnitude of serum cytokine concentrations in hospitalized versus community COVID-19 cases during the first and second week of illness. Cytokine concentrations (pg/mL) in sera were compared between community (gray) and hospitalized (red) study participants at <6 DPOS or 8-12 DPOS, which represent the virologic and immunologic phases of disease respectively. **(A)** In the <6 DPOS time period, IL-6 was significantly higher in hospitalized (n = 27) versus community (n = 22) COVID-19 cases. At 8–12 DPOS, **(B)** IL-6, **(C)** IL-1R antagonist, **(D)** IP-10, and **(E)** MIG were all significantly higher in the hospitalized (n = 33) versus community (43) COVID-19 cases. Mann–Whitney tests with Bonferroni multiple test correction (p < 0.001).

We then asked whether the directionality of cytokine dysregulation was related to severe COVID-19 by comparing community versus hospitalized groups (**Supplementary Figure S1**). We calculated the mean log_10_ fold difference in cytokine level relative to healthy individuals, where above the mean of healthy indicated an upregulation and below the mean indicated a downregulation (**Figure 1**). Some hospitalized patients received antiviral or immunomodulatory medications for COVID-19 or participated in therapeutic clinical trials (**Supplementary Table S2**), whereas the community group did not receive any recorded clinical treatments. We found that IL-8, IL-6, and IP-10 were the most highly upregulated in all COVID-19 community and hospital groups relative to healthy participants, indicating that their upregulation is a hallmark of all SARS-CoV-2 infections (**Figure 2**). However, IL-2 and GM-CSF were upregulated in the community and downregulated in the hospital group at <6 DPOS, suggesting that their early upregulation may be involved in milder disease trajectories (**Figure 1**). Also, IL-17 was upregulated in the hospital group but downregulated in the community group at 8–12 DPOS, suggesting that it may have a role in severe pathology (**Figure 1**).

Next, we compared shifts from the first (<6 DPOS) to second week (8–12 DPOS) of illness. In the community group, mean levels (log_2_ pg/mL) of GROα, IL-1R antagonist, IP10, and MCP1 were significantly lower in the second week (n = 43) compared to the first week (n = 22; Mann–Whitney test, unadjusted p < 0.05; **Table 2**). In contrast, in the hospital group, GROα, IL-1R antagonist, and MCP1 were at similar levels, and only IP10 was significantly lower in the second week (n = 33) compared to those of the first week (n = 27; Mann–Whitney test, unadjusted p < 0.05; **Table 2**). This suggests that prolonged GROα, IL-1R antagonist, and MCP1 may be involved in COVID-19 pathology.

### 3.4 Differential magnitudes of cytokines by hospitalization

To define whether the magnitude of cytokines during COVID-19 differed with severity, we tested differences in mean cytokine levels in community versus hospital groups (*post hoc* Mann–Whitney test with Bonferroni multiple test correction, α < 0.001; **Supplementary Figure S1**). We found that IL-6 was significantly higher in the hospitalized group at both <6 DPOS and 8–12 DPOS than in the community group (p < 0.001; **Figures 2A, B**). Also, IL-1R antagonist, IP-10, and MIG were significantly higher in the hospitalized group at 8–12 DPOS than in the community group (p < 0.001; **Figures 2C–E**). In contrast, the community group elicited 2- to 10-fold lower levels of the same cytokines at <6 DPOS, which further decreased by 8–12 DPOS (**Table 2**). Thus, we found that an early and high level of IL-6 as well as the maintenance of high levels of IL-6, IL-1Ra, IP10, and MIG through 8–12 were related to hospitalized outcome.

### 3.5 Contributions of groups of cytokines toward hospitalization

**Figure 3:**
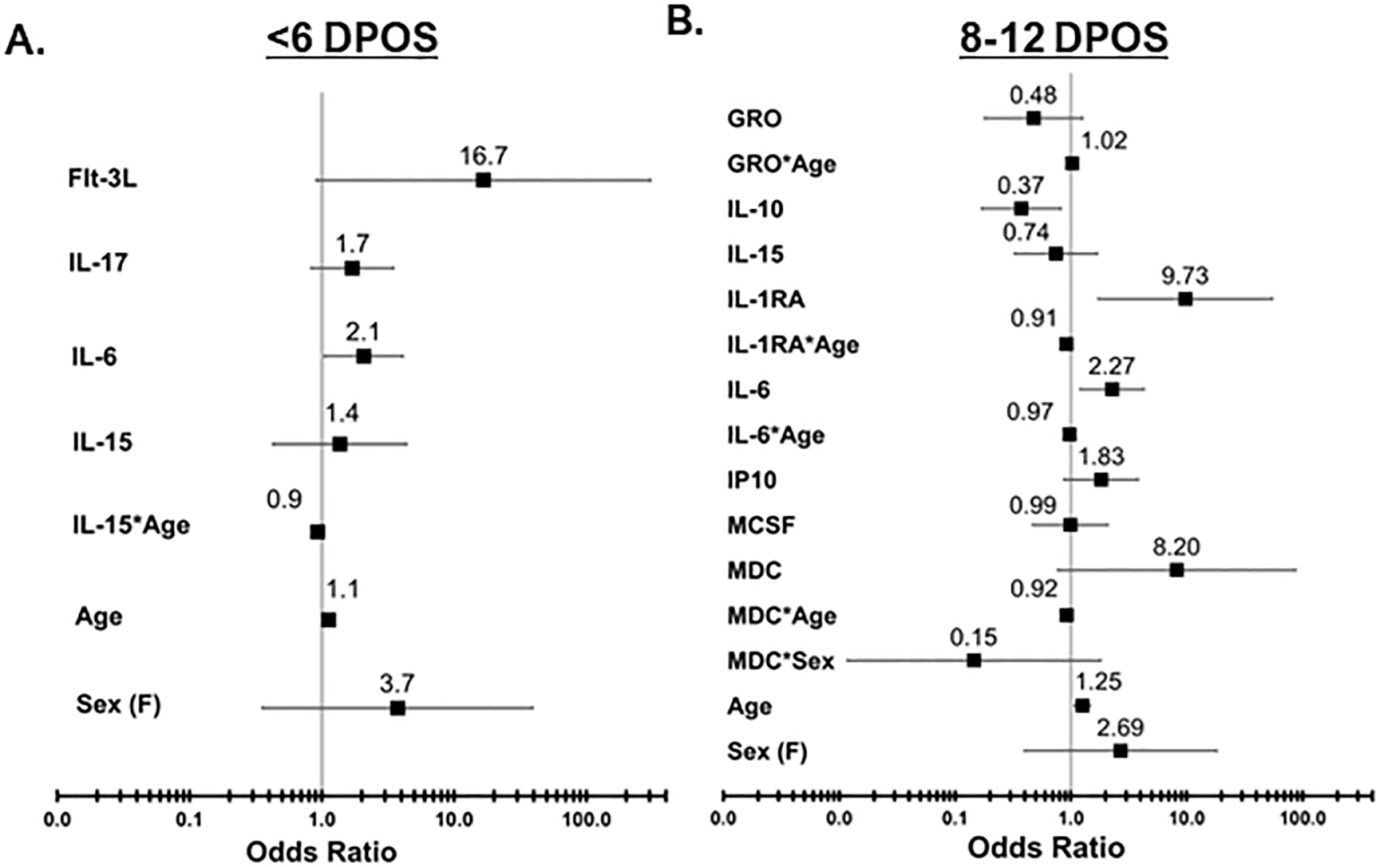
Odds of increased hospitalization is associated with distinct sets of cytokines at <6 vs. 8–12 DPOS during COVID-19. Logistic regression models were used to determine a minimal set of cytokines that together best explains variation in the outcome of hospitalization due to COVID-19, after controlling for age and sex. **(A)** In the <6 DPOS period, FLT3, IL-17, IL-6 (p < 0.05), and IL-15 were associated with hospitalization due to COVID-19 (community group n = 22, hospital group n = 27). **(B)** In the 8–12 DPOS period, GROα, IL-10, IL-15, IL-1RA, IL-6, IP-10, M-CSF, and MDC were associated with COVID-19 hospitalization (community group n = 43, hospital group n = 33). Odds ratios (OR) are presented as solid black boxes, and extended lines indicate their 95% confidence interval. An OR greater than 1 indicates increased likelihood of being in the hospital group than in the community group. If the confidence interval of the OR does not overlap with the null (i.e., 1), then the association of the cytokine with hospitalization is considered significant (p < 0.05). The underlying odds for each hospital and community group were calculated relative to 45-year-old males with average levels of cytokines observed in healthy individuals.

Since cytokines operate in concert with each other to shape downstream immune responses, we defined minimum sets of cytokines that contribute to increased odds of hospitalization at each <6 and 8–12 DPOS using logistic regression models (**Figure 3**). We directly compared the hospital versus community groups, by testing whether a twofold increase in serum cytokine concentrations was associated with a significantly greater likelihood of being in the hospital group than the community group using odds ratio (OR). We also assessed for interaction effects to test whether the relationship of each cytokine with hospitalization depended on either sex or age. At <6 DPOS, a model with FLT3, IL-17, IL-6, IL-15 emerged as the minimal cytokine set that explained 62% of variability in COVID-19 hospitalization (Akaike information criterion or AIC = 41.4; n = 49; **Figure 3A**). Specifically, a twofold increase in the level of IL-6 was significantly associated with two-times greater odds of hospitalization relative to the community group [OR = 2.07 (95% CI = 1.04–4.09), p < 0.05]. At <6 DPOS, higher age, female sex, and higher levels of FLT3, IL-17, and IL-15 were not significantly associated with hospitalization in our study participants (**Figure 3A**). While IL-15 may be slightly related to reduced odds of hospitalization with increasing age, this association was not significant.

In the 8–12 DPOS period, a larger combination of cytokines as well as age- or sex-dependent effects were required to optimally explain 60% of variability in hospitalization (AIC = 72, n = 76; **Figure 3B**). We identified that a twofold increase in IL-10 was significantly associated with a 63% reduction in odds of hospitalization, relative to the community group [OR = 0.37 (95% CI = 0.16–0.81), p < 0.05; **Figure 3B**], whereas a twofold increase in IL-6 [OR = 2.26 (95% CI = 1.2–4.2)] and IP10 [OR = 1.82 (95% CI = 0.87–3.8)] was significantly associated with 1.8- to 2.2-fold increased odds of hospitalization (p < 0.05; **Figure 3B**). A twofold increase in IL-1R antagonist led to a significant 10-fold increase in the odds of hospitalization relative to the community group [OR = 9.7 (95% CI = 1.7–54], p < 0.05; **Figure 3B**). However, IL-1R antagonist levels also demonstrated a small but significant association with reduced odds of hospitalization in higher age groups [OR = −0.09 (95% CI = −0.16 to −0.02), p < 0.05; **Figure 3B**]. Meanwhile, an increase in age significantly raised odds of hospitalization in the second week of illness by 16% in comparison to the community group [OR = 1.25 (95% CI = 1.07–1.46), p < 0.05; **Figure 3B**]. Female sex was not significantly associated with the hospital group. While it is possible that MDC and IL-6 may slightly reduce the odds of hospitalization in older age groups and females, and GROα may slightly increase the odds of hospitalization in older age groups, these associations were not significant. Both models revealed that distinct groups of cytokines are related to odds of hospitalization in the first versus second week of COVID-19 and explain more variability in the odds of hospitalization than age and sex only (AIC = 137–161; n = 129–145).

### 3.6 Early antibody responses upon SARS-CoV-2 infection related to low viral load

**Figure 4:**
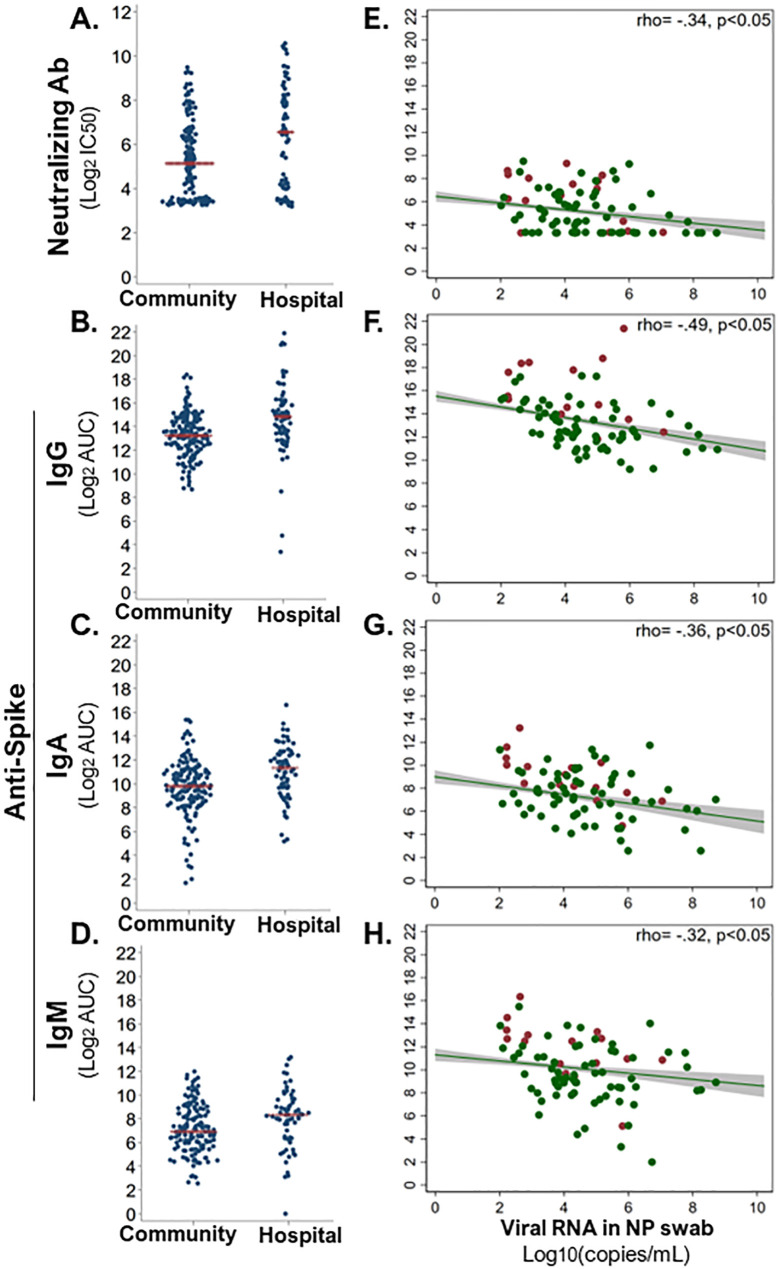
Early antiviral antibody responses in relation to hospitalization and nasal viral load. Serum-neutralizing and virus-binding antibodies from the enrollment sample were compared across community (n = 68–73) and hospital (n = 148–153) COVID-19 cases. **(A)** Serum antibody neutralization of SARS-CoV-2 (strain WA1) was assessed as the titer at 50% maximal infectivity relative to a virus-only control in a focus forming assay (IC_50_). SARS-CoV-2 spike (S)-binding IgG **(B)**, IgA **(C)**, and IgM **(D)** were measured in an ELISA with immobilized antigen and serial dilutions of serum. Magnitude of each binding antibody population was assessed as log_2_ of the area under the curve (log_2_AUC). All antibody classes were significantly higher in hospitalized than in community COVID-19 cases (Wilcoxon signed-rank test, p < 0.05). Red line indicates the mean of each subgroup. Each neutralizing **(E)** and S-binding IgG **(F)**, IgA **(G)**, and IgM **(H)** antibody level at enrollment was significantly inversely correlated with viral load (log_10_ copies/ml) in nasopharyngeal (NP) swab at enrollment (Spearman rank correlation, p < 0.05). Red dots indicate hospital group, and green dots indicate community group. A linear best fit line with 95% confidence interval; Spearman rank rho displayed per plot.

Antibodies that bind viruses can target the virus for destruction, whereas the subset of antibodies that neutralize the virus can prevent infection of host cells. So, we assessed how the magnitude of early SARS-CoV-2-binding and -neutralizing antibody responses differed in community (n = 120–153) versus hospitalized (n = 61–73) SARS-CoV-2 infections. Surprisingly, the hospital group demonstrated significantly greater SARS-CoV-2-neutralizing antibody, and binding IgG, IgM, and IgA responses against SARS-CoV-2 spike (S) and nucleoprotein (NP) than the community at enrollment (Wilcoxon signed-rank test, p < 0.05; **Figures 4A–D** and **Supplementary Figures S2A–C**). This persisted through the first 40 days from symptom onset, such that peak antibody level within 40 DPOS was significantly higher in the hospital versus community group for neutralizing antibodies, S-binding IgG and IgA, and NP-binding IgG, IgA, and IgM (**Supplementary Table S2D**). In each community and hospital group, males generally demonstrated higher SARS-CoV-2-binding antibody levels than females, and antibody levels increased with age from <40, to 40–60, and >60 years of age (**Supplementary Figure S3**). To explain higher antibody levels in more severe cases, we further probed the protective effects of antibodies and asked whether high SARS-CoV-2 antibody responses were related to low early viral burden in the respiratory tract. To test this, we examined the relationship between viral load in the nasopharyngeal swab at the first visit versus serum antibody levels (**Figures 4E–H**). We found that S-binding-IgG, -IgA, and -IgM, as well as neutralizing antibody levels from the first visit were negatively correlated (rho −0.49 to −0.32) with viral load (Spearman rank correlation, p < 0.001). Thus, high early SARS-CoV-2 antibodies were related to lower early viral burden, but not hospitalization.

### 3.7 Longitudinal kinetics of SARS-CoV-2 antibodies in the community

**Figure 5:**
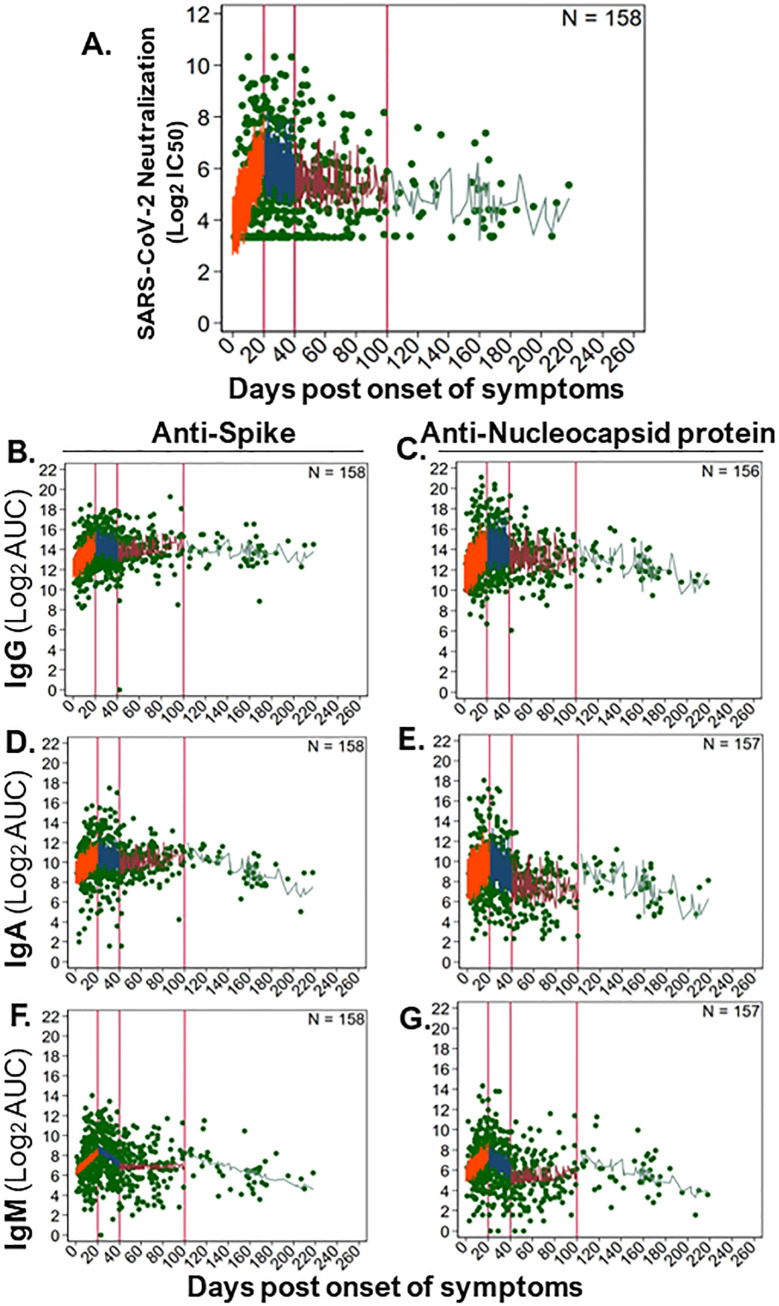
Longitudinal kinetics of SARS-CoV-2-neutralizing and -binding antibodies in the community. To examine long-term dynamics of antiviral antibodies upon natural SARS-CoV-2 infection in community participants (n = 157–158), we analyzed antibody levels over time (586–594 samples) using a generalized linear mixed effects model. Levels of binding antibodies were evaluated as log_2_ of the area under the curve (log_2_AUC) and neutralizing antibodies as log_2_ of the serum titer demonstrating 50% of maximal infectivity (log_2_IC_50_). The following serum antibody populations were examined: **(A)** SARS-CoV-2 (strain WA1) neutralizing antibody titers; anti-spike protein-binding **(B)** IgG, **(D)** IgA, and **(F)** IgM; anti-nucleocapsid protein-binding **(C)** IgG, **(E)** IgA, and **(G)** IgM. The model fit for each antibody subset is shown as a line in each panel, where antibody levels were estimated for small timeframes and demonstrate the variability inherent in our data and sampling. Distinct rates of change in antibody levels were estimated for immunologically relevant time periods, which are separated by vertical red lines. The model fit line for each time period was colored: 0–20 DPOS (orange), >20–40 DPOS (blue), >40–100 DPOS (red), and >100 DPOS (turquoise). Each dilution was measured in replicate. The number of individuals sampled is shown as “N,” and the raw data for antibody level per sample is shown with green points. Linear rates of change in antibody level per DPOS (Δ log_2_AUC/DPOS) were estimated based on these models (see **Supplementary Figure S4**).

Due to a longer follow up of 156 community participants, we were able to evaluate SARS-CoV-2 antibody kinetics up to 6 months post symptoms. We modeled the dynamics of antibody responses as linear rates over four time bins and found that each time bin had distinct rates, as characterized by a steep rise (orange; ≤20 DPOS), then a short and steep decline (blue; >20–40 DPOS), then a period of stabilization (red; >40–100 DPSO), and finally a long-term steady state (green; >100 DPOS; **Figure 5** and **Supplementary Figure S4**). Rates were calculated as twofold change in antibody level for each day post symptom onset (**Figure 5** and **Supplementary Figure S4**). At <20 DPOS, neutralizing antibodies, anti-S IgG, and anti-NP IgG rose faster (rates = +0.12 to +0.15) than antiviral IgA and IgM antibodies (rates = +0.09 to +0.11; **Supplementary Figure S4**). In the >20–40 DPOS period, neutralizing antibodies, antiviral IgG, and anti-S IgA declined at a slower rate (+0.0008 to −0.03) than antiviral IgM and anti-NP IgA (rate = −0.06 to −0.07; **Supplementary Figure S4**). Most antibody populations underwent a slight increase during the >40–100 DPOS period, including antiviral IgA and IgM, neutralizing antibodies, and anti-S IgG (rates= +0.01 to +0.07; **Figure 5** and **Supplementary Figure S4**). Importantly, the longest-term follow-up of >100 DPOS revealed the presence of long-lived antiviral IgM and IgA antibodies, as well as a stable rate for neutralizing and long-lived anti-S IgG antibodies (rate = +0.005 to −0.014; **Figure 5** and **Supplementary Figure S4**).

## 4 Discussion

In this study, we identified that hospitalization due to COVID-19 was characterized by high levels of IL-6, MIG, IP-10, and IL-1R antagonist in the second week of illness and high levels of IL-6 in the first week of illness in comparison to milder illness in the community. Uniquely, we defined distinct cytokine trajectories for severe versus mild disease, which suggests that temporally limited changes in IL-2 and GM-CSF levels in the first but not second week of illness may modulate immunity toward protection. Also, we determined that prolonged high levels of IL-17, and insufficient downregulation of GROα and MCP1 into the second week of illness, may modulate immunopathology associated with disease severity. Our data show that high early neutralizing antibody responses were related to low nasal viral load but not necessarily protection from a more severe disease characterized by hospitalization. We further defined four distinct phases of antibody dynamics, which reveal stable levels of binding and neutralizing antibodies after 100 DPOS. Thus, we defined temporally distinct features of early cytokine and antibody responses to SARS-CoV-2 infection that are associated with severe disease and quantified long-term antibody immunity in the community.

We identified 28 serum cytokines that were differentially expressed in SARS-CoV-2 infection relative to healthy individuals within 2 weeks of symptoms, including high levels of IL-6, IL-8, and IL-10, which align with prior studies (21, 44–52). Specifically, the magnitudes of circulating IL-6, IL-1R antagonist, MIG, and IP-10 in the second week of illness were significantly higher in the hospital than in community cases, supporting the role of these cytokines in pathology (44–46, 50–53). IL-6 is a secreted molecule that can activate B- and T-cell function, recruit monocytes and neutrophils, and increase inflammation and coagulation (54, 55). Interestingly, IL-6 shares a common pathway with the other cytokines identified here such as IL-1, which in turn can stimulate production of IP-10 and MIG to amplify signals by interferon γ and recruit immune cells (55, 56). Also, MIG may be secreted by T cells and monocytes and serves to recruit T cells (57). The functions of these cytokines correspond to COVID-19 pathology as evidenced by macrophage, neutrophil, and T-cell infiltrates in lung tissue and high coagulation proteins in the blood of patients (11, 47, 58). While the initial nasal viral load was no different by hospitalization, serum cytokine levels in hospitalized cases deviated more from healthy levels than in community cases reinforcing the hypothesis that greater cytokine dysregulation underlies pathology (21, 44–52). Indeed, immunomodulators, like IL-6 inhibitors (tocilizumab or sarilumab), were found to reduce mortality in severely ill patients (21, 44–52, 59, 60). These data support the conclusion that greater cytokine dysregulation, particularly IL-6, is associated with hospitalization due to severe COVID-19.

Inference of cytokine trajectory through analysis of cytokine levels in the first versus second week of illness revealed distinct temporal dynamics that may underlie progression to severe disease. First, the pro-inflammatory cytokine IL-17 was ninefold higher in the second week of illness than in the first week in the hospital group, but remained constant in the community group, supporting that IL-17 is involved with immunopathology (26, 61, 62), although similarly high levels of IL-17 in acute common cold coronavirus infections are typically not associated with severe outcomes. This may be because the pathologic effects of IL-17 depend on the timing and milieu of other cytokines which are also high during SARS-CoV-2 but not in common cold coronavirus infections (16). Second, serum IL-2 and GM-CSF were elevated in the community group during the first week of illness and then returned to healthy group levels by the second week. But these cytokines remained low in the more severe hospital group. This suggests that early increases in IL-2 and GM-CSF followed by rapid regulation of their levels may be involved in cultivating effective and self-limiting antiviral immune responses. Since GM-CSF is a growth factor for granulocytes and macrophages, and IL-2 can stimulate T cells, they may serve to improve lung oxygenation and clear virus (16, 45, 54, 63, 64). Third, serum GROα, IL-1R antagonist, and MCP-1 were high in both the first and second weeks of illness in the hospital group but decreased over time in the community group supporting that their prolonged high circulating levels are associated with severity (21, 44, 52). These cytokines recruit monocytes and neutrophils, which may initially clear virus but later cause tissue damage (55). Thus, there may be a time-limited role for IL-17, IL-2, GMCSF, GROα, IL-1R antagonist, and MCP-1 in shaping protection versus pathology. Future studies should incorporate temporally distinct investigation of cytokine immunity and validate these trajectories through longitudinal sampling.

Given the pleiotropic, redundant, and cooperative functions of cytokines, we identified sets of cytokines that may work together to elicit immune phenotypes associated with hospitalization (16, 65). In the first week of illness, increased in levels of IL-6, FLT3, IL-17, and IL-15 are associated with increased odds of hospitalization, while each of these cytokines were previously found to be associated with severity, and our model suggests they may work together (21, 44–47, 61). Biologically, FLT3 responds to IL-6 and promotes differentiation of progenitor immune cells but can also increase platelet count, which is a common pathology of COVID-19 (66). The approval of baricitinib as an effective treatment for severe COVID-19 concurs with our finding because this intervention disrupts FLT3 function by inhibiting JAK signaling (65). Also, IL-6, FLT3, and IL-15 can all cause excess T-cell stimulation leading to induction of IL-17-secreting T cells, which are known to infiltrate lung tissue and diminish oxygenation in severe COVID-19 (54). Thus, it is possible that these cytokines together promote T-cell-mediated pathology in COVID-19. Their interactions should be further investigated and harnessed for improved targeting of immunomodulatory therapies (65, 67–69). Given high inter-individual variability in clinical outcomes upon IL-6 or JAK/STAT inhibition therapies, the activity of this pathway in hospitalized patients may help to predict who will respond to therapy and can guide personalized medicine approaches (16, 65).

The second week of illness revealed more complex associations of cytokines with severity that were harder to deconvolute because of heterogeneity in COVID-19 immunopathology. Increased odds of hospitalization were associated with cytokines that promote inflammasome activation (IL-1R antagonist), interferon-γ signaling (IP-10), positive feedback of IL-6 production, recruitment of neutrophils and granulocytes (GROα and IL-15), and activation of macrophages (MDC and M-CSF) (54, 55, 70–73). In contrast, higher levels of the anti-inflammatory cytokine IL-10 demonstrated a slightly protective effect. Interestingly, our models posit that GROα, IL-1R antagonist, and IL-6 may contribute age-dependent effects, and this is supported by prior studies as older age modulates expression of these cytokines (74, 75). This is compatible with high inflammatory markers, cytotoxic immune cells, and macrophages as key players in the second week of illness during severe COVID-19 (23, 73, 76).

We then investigated antibody responses in community versus hospitalized participants. While SARS-CoV-2 neutralizing antibodies are thought to be protective, we paradoxically found higher levels of binding and neutralizing antibodies in serum samples from the first visit of hospitalized cases compared to community participants, like a prior study (45). One explanation for this is that the earliness of antiviral antibodies matters in limiting infection (32). In support of this, we found that high early antibody levels were correlated with lower nasal viral load at that same early timepoint, even though they were not different by group. Thus, our data are compatible with an early role for antiviral antibodies in controlling infection.

Waning antiviral antibodies have been a concern throughout this pandemic and guided requirements for booster vaccinations (34, 77, 78). We quantified antibody dynamics in mild community infections and found peak neutralizing and binding antibody responses by 3 weeks post symptom onset, and they remained detectable up to 6 months post infection; this concurs with previous studies (79, 80). While IgM and IgA isotype antibodies are thought to be short-lived, we detected antiviral IgA and IgM antibodies up to 6 months post infection (81). The long-term presence and functions of these antiviral IgM and IgA subsets should be further evaluated.

Interestingly, our work identifies four distinct phases of antibody kinetics upon SARS-CoV-2 infection and shows that the early waning kinetic within 100 days of symptoms does not reflect the long-term steady state beyond 100 days. This suggests that some of the early models of antibody waning kinetic may be overly conservative in their estimates and that SARS-CoV-2 antibodies may be maintained at a long-term low-level steady state like childhood vaccines and other viruses where antibody data are available for years (77, 80, 82–84).

Our study has several notable strengths. First, in contrast to most studies on subsets of hospitalized patients, we profiled milder responses to infection by extensive sampling of community members in their residence. This fills a gap on benchmarking non-pathological and protective immune responses to COVID-19. Second, we accounted for days post symptom onset as a proxy for time as immune responses are inherently dynamic. Third, we conducted a comprehensive analysis of 47 cytokines at two immunologically relevant timepoints, 6 antiviral binding antibody populations, and neutralization of the actual WA-1 virus strain (not a pseudo virus or binding assay proxy) from 158 participants’ samples. These strengths allowed us to identify temporally distinct cytokine trajectories that may underlie disease severity and quantify longitudinal antibody immunity.

Our study has some limitations. First, our statistical analysis of circulating cytokine is based on associations with outcomes and does not indicate causal effects. Second, we used hospitalization as a proxy for disease severity, and our hospital group was highly heterogenous compared to prior studies on subsets of severe hospitalized COVID-19 patients because this study was performed at a time when individuals with age or comorbidity were admitted regardless of disease severity. Thus, some cases may have been hospitalized without severe symptoms due to public health necessity (i.e., residents of nursing homes), which was particularly relevant early in the pandemic. Use of hospitalization as a comparator group was intentional as our goal was to understand how community infections differ from the undesirable outcome of hospitalization. Third, our antibody kinetic analyses are limited to the community group because we were unable to consistently collect long-term samples from the hospitalized group. Fourth, although recent studies reveal that immune responses triggered by different SARS-CoV-2 variants can vary (85), we were not able to perform additional sequencing to conduct a stratified analysis by viral variant. However, as a temporal proxy, our study period occurred during the alpha and delta variant periods, and not the omicron variant, which has substantially impacted immunity.

In conclusion, we find that cytokine dysregulation is associated with development of severe COVID-19 as represented by hospitalization. While elevated early anti-SARS-CoV-2 antibodies were associated with low nasal viral loads, they did not correlate with avoiding hospitalization. Indeed, breakthrough infections after vaccination were not only characterized by the presence of early neutralizing antibodies but also lower levels of cytokine responses than natural infection, which may underlie protection from severe disease (44). Also, our study suggests a protective role for some cytokines in a time-limited or level-specific manner underscoring the need to understand each patient’s immunopathological progression for effective intervention with personalized immunotherapies. Finally, our community participants’ data provide a benchmark for cytokine profiles in mild natural infections that were successfully resolved, and this will be valuable to further optimize immunomodulatory therapies.

## Data Availability

The raw data supporting the conclusions of this article will be made available by the co-corresponding authors, without undue reservation.
