# Supplementary material for: Dynamics of cytokine and antibody responses in community versus hospital SARS-CoV-2 infections
Source: Front Immunol. 2024 Nov 22;15:1468871. doi: 10.3389/fimmu.2024.1468871 (PMC11621060; doi:10.3389/fimmu.2024.1468871)
Supplement: Supplementary file 1 [file DataSheet1.docx]

# **Supplementary Materials**

**Dynamics of cytokine and antibody responses in community versus hospital SARS-CoV-2 infections**

**Tulika Singh, Andrew N. Macintyre, Thomas W. Burke, Jack Anderson, Elizabeth Petzold, Erica L. Stover, Matthew J. French, Thomas H. Oguin III, Todd Demarco, Micah T. McClain, Emily R. Ko, Lawrence P. Park, Thomas Denny, Gregory D. Sempowski, Christopher W. Woods**

**Table S1. Characteristics of COVID-19 cases sampled in prospective cohort of community and hospital infections in North Carolina from March 2020 – November 2021.**

**
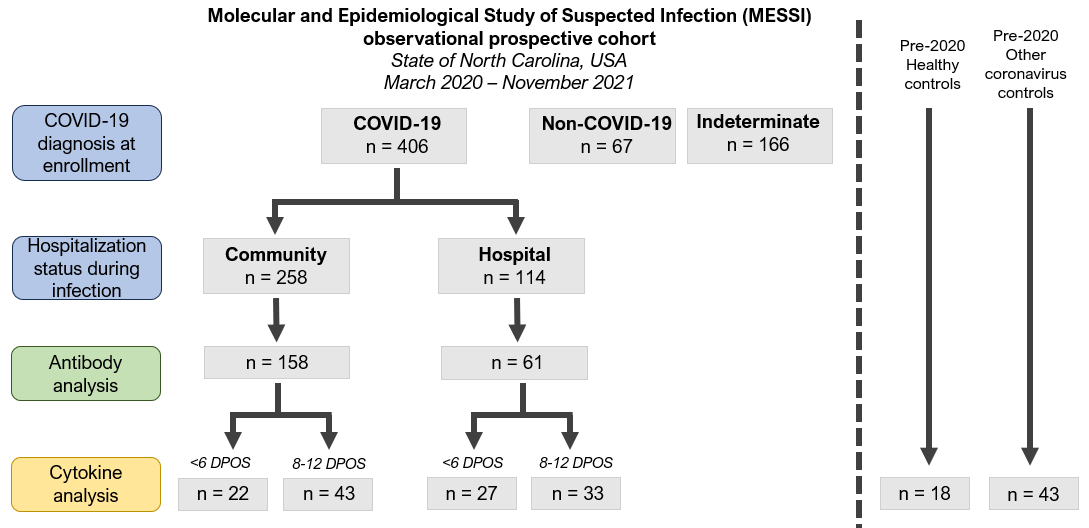
**

**Fig S1. Observational study design indicating selection of key comparator groups as community versus hospital COVID-19 infections.** From March 2000 to November 2021, community and hospitalized individuals were enrolled into a prospective observational cohort and longitudinally sampled. Of these, 406 were verified as SARS-CoV-2 infections (indicated as COVID-19). From this, a subset of 258 individuals were never hospitalized during their infection and/or illness (i.e. community group), and 114 others were treated in the hospital during their infection. From these groups, a convenience sample was taken for assessment of antibody and cytokine responses. In particular for cytokine responses, samples were selected within <6 or 8-12 DPOS time bins to study temporally relevant cytokine immunity. Pre-pandemic samples (right of dotted line) from either healthy infections were used as negative controls for the cytokine analysis. N indicates the number of people and downward arrows define subsets.

**Table S2. Steroid treatments or interventions given to hospitalized COVID-19 individuals included the cytokine analysis of this study.**

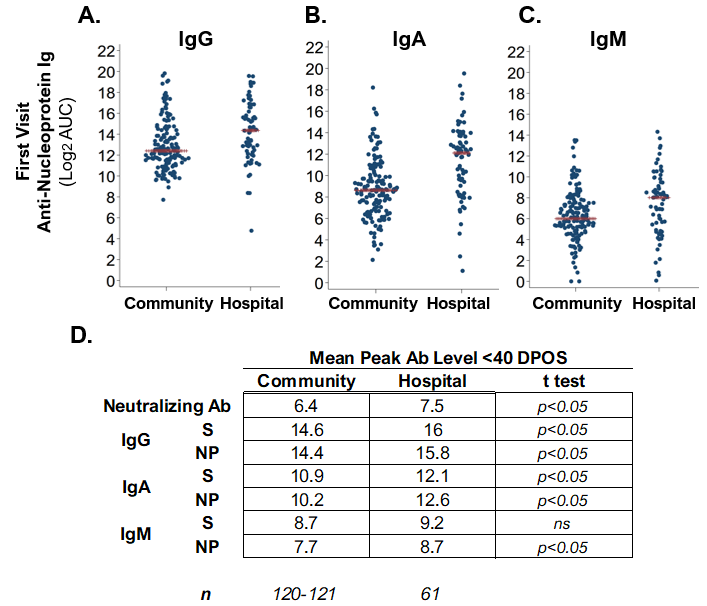


**Fig S2. Early and peak antibody responses in community versus hospital COVID-19 cases.** The magnitude of nucleoprotein (NP) binding IgG **(A)**, IgA **(B)**, and IgM **(C)** from the enrollment sample were compared across community (n=68-73) and hospital (n=148-153) COVID-19. Each binding antibody population was significantly higher in hospitalized as compared to community COVID-19 cases (t-test, p<0.05). **D.** The peak level of neutralizing, S-binding or NP-binding antibody within 40 days of symptoms was compared for the community versus the hospital group. The hospital group demonstrated higher peak levels of antibodies within 40 days of symptoms than community COVID-19 cases (p<0.05, t-test). Serum antibody neutralization of SARS-CoV-2 (strain WA1) was assessed as the titer at 50% maximal infectivity relative to a virus only control in a focus forming assay (IC_50_), and S or NP binding antibodies were assessed via serial dilution on an ELISA and quantified as Log_2_ of the area under the curve (Log_2_AUC).


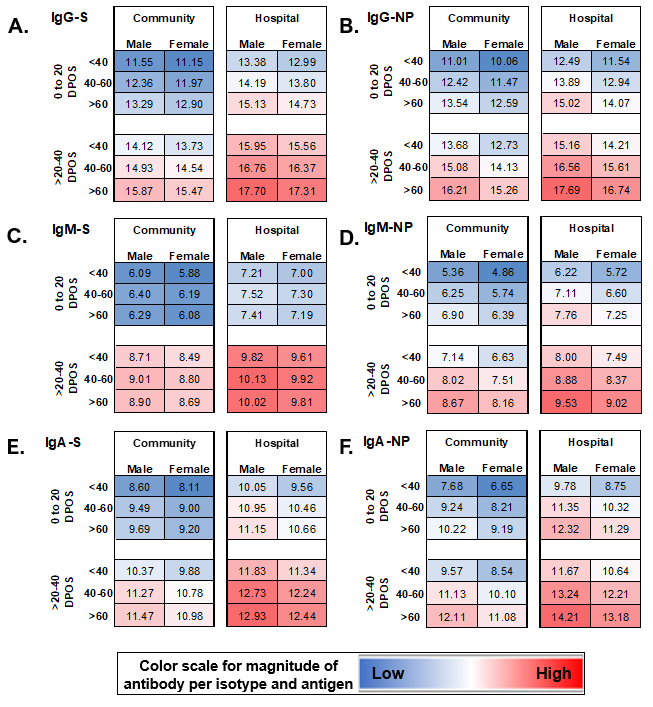


**Fig S3**. **Early virus binding antibody levels are highest in hospitalized cases of SARS-CoV-2, males, and older adults.** A generalized estimating equation model was used to estimate the relationships between the levels of each virus binding antibody population and subsets of SARS-CoV-2 infections among people with different age, sex, and hospitalization status. The main comparisons are between community (participants n=149, participant-timepoints n=498) versus hospitalized (participants n=66, participant-timepoint n=136) SARS-CoV-2 infections. Antibody levels are shown as means of Log_2_ transformed Area Under the Curve (Log_2_AUC) of a serial dilution of serum tested for binding with spike protein (S) or nucleocapsid protein (NP). S and NP binding antibodies were measured for each antibody isotype: IgG, IgM, and IgA. Mean antibody levels for each group of people were estimated for S-binding IgG **(A)**, IgM **(C)**, and IgA **(E)**, as well as NP-binding IgG **(B)**, IgM **(D)**, and IgA **(F)**. Each antiviral antibody type was assessed for each 0-20 DPOS and >20-40 DPOS time bins to delineate patterns over time. The contribution of age was assessed in categories of <40, >40-60, and >60 years. Color gradients denote levels of antibodies within each subset (i.e. panel), where red is a high level of antibody and blue is a low level of antibody.


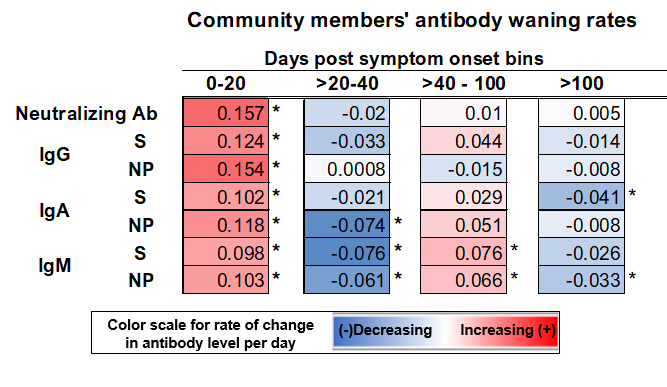


**Figure S4. Estimated antibody waning rates in the community COVID-19 group by immunologically relevant time periods since symptom onset.** Change in antibody level per day was quantified as rates: the two-fold (Log_2_) change in area under the curve (AUC) for binding antibodies in serum or two-fold (Log_2_) change in 50% maximal neutralizing antibody titer (IC_50_) for every one-day post symptom onset (Δ Log_2_ Antibody / DPOS). Rates were estimated for each time period: 0-20 DPOS, >20-40 DPOS, >40-100 DPOS, and >100 DPOS. Color denotes the rate of change in levels of antibodies per day such that a positive value (red) indicates an increase in antibody level over time and a negative value (blue) indicates a decrease in antibody level over time. The further the value from 0, the greater the rate of change in the level of antibody (color intensity). In total, data from 157-158 community participants and their 586-594 samples are included in this model. S denotes spike protein binding antibody and NP denotes nucleocapsid binding antibody for which IgG, IgM and IgA isotype subsets were measured.

**Table S3. List of equations applied for analysis referenced in the Methods Sections on logistic regression and statistical models.**

| **Eq 1** | $E\left[ Y \right]= {}_{0}+ {}_{1}(C_{1})+ e$ |
| --- | --- |
| **Eq 2** | $E\left[ Y \right]= {}_{0}+ {}_{1}(C_{1})+{}_{2}(age)+{}_{3}(sex)+ e$ |
| **Eq 3** | $E\left[ Y \right]= {}_{0}+ {}_{1}\left( C_{1} \right)+{}_{2}\left( age \right)+{}_{3}\left( sex \right)+{}_{4}\left( age\times C_{1} \right)+{}_{5}\left( sex\times C_{1} \right)+ e$ |
| **Eq 4** | $E\left[ Y \right]= {}_{0}+ {}_{1}\left( FLT3L \right)+{}_{2}\left( IL17 \right)+{}_{3}\left( IL6 \right)+{}_{4}\left( IL15 \right)+{}_{5}\left( age\times IL15 \right)$  $+ {}_{6}(age)+{}_{7}(sex)+ e$ |
| **Eq 5** | $E\left[ Y \right]= {}_{0}+ {}_{1}\left( GROa \right)+{}_{2}\left( IL10 \right)+{}_{3}\left( IL6 \right)+{}_{4}\left( IL15 \right)+{}_{5}\left( IL1Ra \right) +{}_{6}\left( IP10 \right)+{}_{7}\left( MCSF \right) +{}_{8}\left( MDC \right)+{}_{9}\left( age \right)+{}_{10}\left( sex \right)+{}_{11}(age\times GROa)+ {}_{12}(age\times IL1Ra)+{}_{13}(age\times IL6)+{}_{14}(age\times MDC)+{}_{15}(sex\times MDC)+ e$ |
| **Eq 6** | $E\left[ Ab level log2\left( AUC \right) \right]=$  ${}_{0}+{}_{1}\left( age 40-60 \right)+{}_{2}\left( age>60 \right)+{}_{3}\left( female \right)+{}_{4}\left( hospitalized \right)$  $+{}_{5}\left( timebin1 \right)+{}_{6}\left( timebin2 \right)+{}_{7}\left( interval2 \right)+ e$ |
| **Eq 7** | $E\left[ Ab level log2\left( AUC \right) \right]={}_{1}\left( age \right)+{}_{2}\left( gender \right)$  $+ {}_{3}\left( timebin1 \right)+{}_{4}\left( timebin2 \right) + {}_{5}\left( timebin3 \right)+{}_{6}\left( timebin4 \right)$  $+ {}_{7}\left( int1 \right)+{}_{8}\left( int2 \right) + {}_{9}\left( int3 \right)+{}_{10}\left( int4 \right)+ e$ |
